# Addressing the Root Causes of Population Health in Central Appalachian Virginia

**DOI:** 10.13023/jah.0703.03

**Published:** 2025-09-01

**Authors:** David L. Driscoll, Kol Gold

**Affiliations:** Healthy Appalachia Institute, UVA Wise; Healthy Appalachia Institute, UVA Wise

**Keywords:** Appalachia, Intervention Science, SDOH, Virginia

## Abstract

**Introduction:**

The Southwest Virginia Health Authority (SWVHA) recently commissioned a community health needs assessment (CHNA) to reduce the high rate of preventable health problems in Virginia’s (VA) Appalachian Region. The CHNA took place over a two-year period from 2023 – 2025.

**Purpose:**

This iterative, mixed-method CHNA informed the development of a new iteration of the regional *Blueprint for Health Improvement and Health-Enabled Prosperity* identifying and prioritizing health issues and develop strategic planning to address them in VA’s three westernmost regional health districts.

**Methods:**

The CHNA followed a sequential mixed methods design to assess the regional health status, the contextual factors associated with any health disparities identified, and the development of a participatory community health improvement plan to modify those contextual factors. The sequential approach involved three phases: in Phase 1, the study team collected and compiled primarily quantitative secondary data from local and regional sources. These data informed the subsequent collection and analysis of quantitative and qualitative data in Phase 2, and the data from Phase 2 informed development of a collaborative community-based strategic implementation plan in Phase 3.

**Results:**

The all-cause mortality rate for the region is roughly double the state average. Residents are dying at a higher rate, and at younger ages, due to inadequate access to quality health care, educational opportunities, income stability, and treatments for substance use disorder. Community stakeholders recommended interventions to address the combination of access to quality care and rurality, employment/income, trauma, and substance use disorder, and education and nutrition. Based on these priorities, seven local non-profit organizations were selected for implementation funding.

**Implications:**

This iterative effort supported the development of integrated and community-based population health interventions in the region. Future regional community health assessments will apply similar methods to evaluate progress on these projects and recalibrate regional efforts in response to evolving local needs and priorities.

## INTRODUCTION

Residents of Virginia’s (VA) Appalachian Region experience higher rates of preventable health outcomes compared to state and national averages. Appalachian VA’s mortality rates for heart disease and COPD, for example, are 40% and 65% higher than the state average, and the region experiences a 54% higher rate of years of potential life lost than non-Appalachian VA.^1^ There is a paucity of information on the root causes of these health disparities, or how to effectively address them.

In 2007, the Virginia General Assembly created a special state organization to bring area leaders together to recommend ways to improve health and health related prosperity in the far Southwest VA region. This organization was named the Southwest Virginia Health Authority (SWVHA). Today, the SWVHA board of directors includes VA legislators, health service providers, public health district leaders, scholars, and appointed county representatives. The SWVHA has commissioned a series of community health needs assessments (CHNAs) to guide their coordinating role. The results of these CHNAs are entitled *Blueprints for Health Improvement and Health-Enabled Prosperity*.

**Figure 1:**
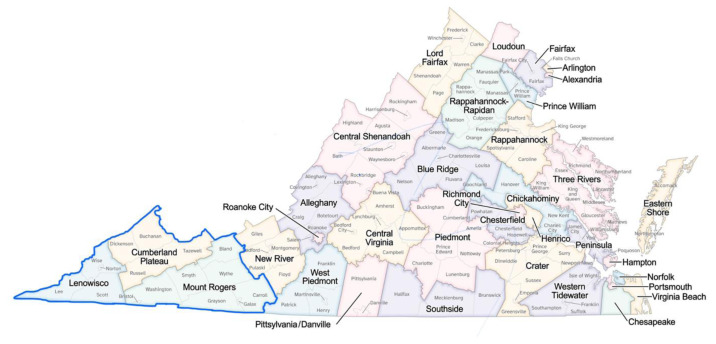
Study Region NOTES: Source: Virginia Department of Health. Blue border inserted.

This paper describes the most recent CHNA, which was focused on three key objectives:

1. Assessing economic and health trends in the 13 westernmost counties of VA, or the Lenowisco, Cumberland Plateau, and Mount Rogers Health Districts (Fig. 1). All these counties are in the Appalachian Region.
2. Identifying the local contextual factors of health disparities and evidence-based programs to address those factors.
3. Developing a strategic plan to prioritize, implement, and evaluate specific evidence-based programs in the study region.

## METHODS

**Figure 2:**
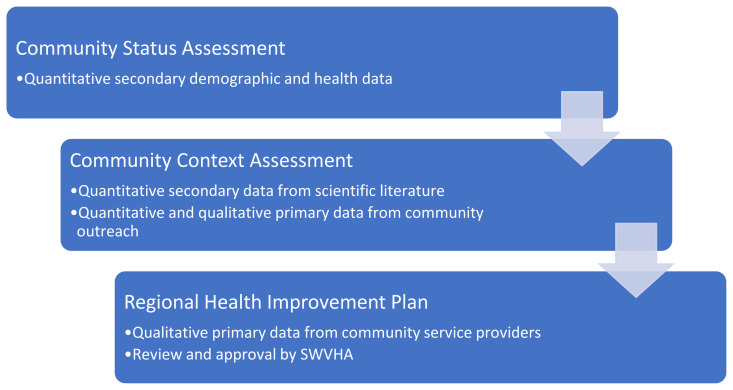
Mixed Method Design

The CHNA followed a sequential mixed-methods research design^2^ (Fig. 2) over a two-year period from 2023 until 2025. The sequential approach involved three phases: in Phase 1 the study team collected and compiled primarily quantitative secondary data from local and regional sources. These data informed the subsequent collection and analysis of quantitative and qualitative data in Phase 2, and the data from Phase 2 informed development of a collaborative community-based strategic implementation plan in Phase 3.

### Phase 1: Community Status Assessment

During the first phase, the study team compiled data from local and state government records and registries over the past ten years to assess regional trends and outcomes. The assessment included demographic and socioeconomic indicators such as population size, geographic distribution, educational attainment, and income level. Specific health metrics included rates and causes of mortality and prevalence of various risk factors such as hypertension, tobacco use, and obesity. To compare regional and statewide years of potential life lost, county-specific data for the study region were collected from the University of Wisconsin Population Health Institute’s County Health Rankings^3^ and then compared to the statewide average.

### Phase 2: Community Context Assessment

In the second phase, the study team identified the social and physical contextual factors contributing to the data collected in Phase 1. The team integrated scientific literature with primary data collected through community-based discussions and surveys. Root causes of these disparities were identified from the University of Wisconsin Population Health Institute’s County Health Rankings, the latest Appalachian Regional Commission (ARC) Report on regional determinants of health,^4^ and archival data from previous *Blueprints*. These root causes served as search terms for a scoping review of Appalachian health literature to assess the latest evidence on population health determinants in Appalachia and to identify research that points to exemplary programs for potential implementation in Central and Southcentral Appalachian regions of Virginia.^5^

The scoping review involved a comprehensive and iterative search by the authors and their partners using the Web of Science (WoS). This citation indexing service provides access to content in the life, biomedical, and social sciences as books, journals, conference proceedings, workshops, and professional reports. The search employed keywords related to Appalachia and the causes of morbidity and mortality in the study region. There were no restrictions placed on language. All searches were conducted from February 1 – April 30, 2023.

The following inclusion criteria were used: (1) original article written by the person or people that conducted the research, (2) published in the last five years (2018 – 2023), (3) involving an Appalachian population, and (4) included a rigorous assessment of an association between a social or environmental determinant and one or more leading causes of Appalachian morbidity and mortality.

All citations were downloaded into Zotero for the initial screening and for the full-text review. Uncertainties were resolved during full team discussions, and the investigators reached a full consensus. The following information from each included study was extracted: (1) related health outcome, (2) type of study, (3) involvement of a pediatric population, (4) testing of intervention, (5) effectiveness of intervention, (6) a brief description of the intervention, (7) corresponding author institution, (8) state(s) where the study was conducted, and (9) applicable population health determinants. The interventional studies were then categorized by program type.

The scoping review findings were then refined through direct engagement with residents at community outreach events. The process began with a meeting of a local strategic advisory group of community stakeholders. Members included public health, clinical, and social service providers who had participated in prior CHNAs. The objective of the meeting was to discuss the scoping review findings and develop next steps for the outreach process. These discussions resulted in two methodological recommendations; first that a snowball sampling methodology be used to identify and engage a broad variety of community residents, and second, that outreach events take place at local events and venues rather than in clinical or academic settings. The only eligibility criterion recommended was participants be residents of, and/or health care providers at organizations serving the 13-county study region residents of the study region.

The study team convened 18 community listening sessions and collected survey data with 272 participants from August – December 2023. Community outreach included unstructured listening sessions and structured surveys conducted at both informal gatherings (e.g., county fairs, fall festivals, mobile clinical service programs) and formal meetings (e.g., at libraries, schools, and community centers). The listening sessions were publicized by local community partners including members of the strategic advisory group and by the study team through flyers and advertisements on social media. Session participants were self-selected. All participants received a complimentary notepad and pen to record their questions and comments during the sessions.

In these unstructured listening sessions, investigators provided a brief presentation of the scoping review findings and responded to comments and questions from residents. Residents’ comments related to the nature and direction of contextual factors contributing to regional health disparities were documented in fieldnotes without session attribution and analyzed using grounded theory analysis. Residents were also invited to complete a structured survey either on paper or electronically. The survey instrument was composed of four categories of ordinal survey questions: the first prioritized community assets and resources, the second the leading community health problems, the third the leading root causes of these health problems, and the fourth preferred programs or services to intervene on these root causes. All categories included open-ended questions for unstructured response categories. A final section consisted of nominal questions related to household income, demographics, and health care coverage and sources. The survey data were also integrated across the study region to ensure respondent anonymity. Survey responses from residents of the study area confirmed by zip codes were entered and analyzed in Qualtrics. The survey instrument and analytic protocol was reviewed and approved by the UVA Social and Behavioral Institutional Review Board in August 2023 (UVA SBS IRB #6052).

### Phase 3: Regional Health Improvement Plan

Findings from Phase 2 were synthesized into a draft document describing the priority health issues in the region, their root causes, and proposed evidence-based strategies found in the scoping review. The document was distributed to a regional advisory group composed of 46 representatives of regional public health agencies, federally qualified health centers, hospital systems, educational institutions, and nonprofit organizations. These stakeholders were asked to assess:

1. Alignment of the proposed strategies with the priorities described by study participants,
2. Perceived feasibility of each strategy in the study region, and
3. Potential to improve regional population health outcomes.

These recommendations were incorporated into a final list of programmatic domains and strategies, including implementation and evaluation plans. They were presented to the SWVHA in April 2024. The presentation was organized into four sections, with each followed by a facilitated discussion:

1. Community status assessment findings and methods.
2. Scoping review methodology and major determinants of regional health disparities.
3. Summary of community outreach activities, including listening session protocols, survey results, and qualitative themes from open discussions.
4. Proposed programmatic domains and strategies, including implementation and evaluation plans.

## RESULTS

### Phase 1: Community Status Assessment

Virginia’s three westernmost health districts have seen a 7% population decline over the past decade, compared to an 8% increase statewide.^6^ Only 16% of residents have a college degree, compared to 41% statewide.^7^ Per-capita income is $46,258 and 19% of the region’s residents live below the federal poverty level, or roughly half and twice the state averages, respectively.^8^

Residents of the region face elevated health risk factors, including higher rates of adverse childhood experiences, depression, elevated cholesterol, blood pressure, smoking, and obesity.^9^–^17^ These risks contribute to a regional all-cause mortality rate of 1,860 per 100,000 compared to a state average of 904 per 100,000.^18^ Aggregated county-specific years of potential life lost rates are 10,998 per 100,000 compared to a state average of 6,707 per 100,000.^19^ The next study phase sought to identify some of the contextual factors creating these health disparities, which are often referred to as the determinants of population health.^20^

### Phase 2: Community Context Assessment

**Figure 3:**
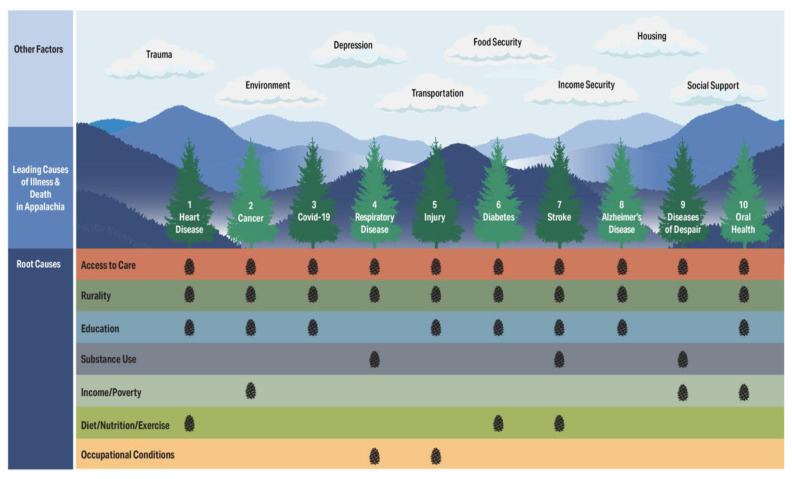
Seeing the Forest: Appalachian Health

The WoS database search and articles identified through other sources returned 2,477 titles and abstracts, of which 476 potentially relevant studies were subjected to full-text review, and 221 (full list available upon request) that rigorously demonstrated an association between one or more determinants of population health in Appalachia and health disparities in our study region. The top five determinants of the region’s leading health disparities were (listed in order of frequency of citations), (1) access to quality health care, (2) rurality, (3) education, (4) employment/income, and (5) substance use disorder. Fig. 3 illustrates the leading causes of illness and death based in incidence data in the 13-county study region as pine trees, with disease determinants from the scoping review depicted as geological strata, ordered by the frequency of citations in the literature. Pinecones mark determinants with an above-average number of associated studies, while additional contextual factors offered by residents during community discussions appear as clouds.

This phase shared the results of our scoping review, including Fig. 3, with community residents to collect deeper insights into these associations and inform targeted interventions. Most of the self-selected sample identified as female (80%), married or with a domestic partner (71%), and white (95%). Most had lived in their community for more than ten years (77%), received health care in a medical provider’s office (85%), and were employed (84%). The participants represented a variety of age categories (25% were under 34, 41% between 35 – 54, and 34% over 54 years of age). Participants had a variety of educational backgrounds (4% had no high school diploma or GED, 15% had graduated either high school and/or vocational school, 20% had some college with no degree, 37% had either an associate or bachelor’s degree, and 24% had a graduate or professional degree). They had a wide range of annual household incomes. Nearly 6% had an annual household income of less than $10,000 annually, 28% earned between $10,000 and $49,999, 38% earned between $50,000 and $99,999, and 27% earned more than $100,000. These higher-than-average levels of education and income were likely due to the large percentage of health professionals participating in the study.

In community discussions of the scoping review findings, many residents expressed dissatisfaction with the term “determinants” of regional population health. Some residents preferred the terms “root causes” or “drivers” to indicate their health outcomes were not predetermined. They also suggested that several of the root causes described in the literature were so intertwined as to be indivisible for the purposes of mitigation or prevention. For example, access to care was defined in the literature by the presence or absence of clinical infrastructure providing preventive, dental, primary, secondary, or tertiary care. Rurality was defined as the variety of geographic, transportation, topographic, economic, and cultural factors that result in isolation and disenfranchisement from social and health care services and programs for rural residents. Residents commented that the presence of clinical infrastructure in a region was insufficient to improve access to care if the factors under rurality were not also mitigated. Residents also highlighted the causal association between regional mental health challenges, including widespread childhood trauma, poverty, and stress from these factors, and the high prevalence of substance use disorder (SUD) in Central Appalachia. They suggested that effective prevention, early intervention, and treatment of SUD should integrate a trauma-informed approach to mental health and resiliency, stigma reduction, social welfare (e.g., housing, food security, transportation), and employment support services.

**Figure 4:**
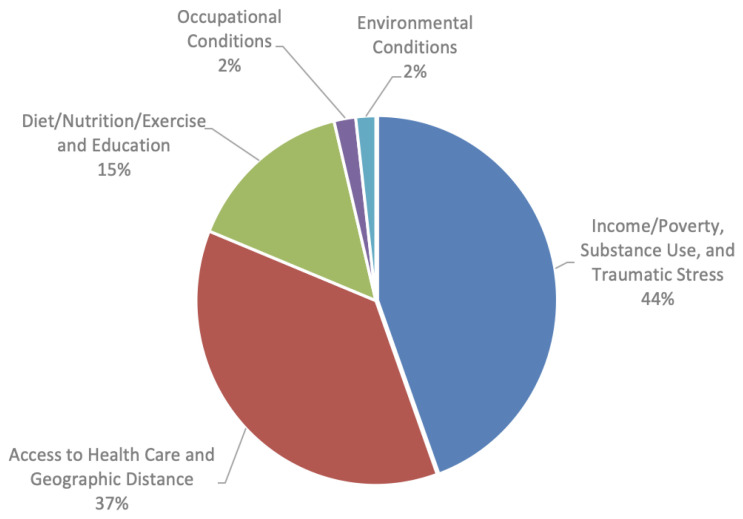
Top Health-Related Concerns in the Community

Survey respondents most frequently reported that the primary assets of the region were the safety and friendliness of their communities. The next most-frequently selected benefit to living in the area was easy access to parks and recreational areas. Respondents listed diabetes, cancer, heart disease, and substance overdose as leading health problems in the region. Respondents were asked to list the priority health concerns, defined as the root causes of the priority health problems. Respondents described several of these concerns as indivisible for the purposes of mitigation or prevention and re-categorized several of these concerns into one domain for intervention. They selected three recategorized priority health-related domains for immediate intervention. The most frequently selected response category was a combination of “Income/Poverty”, “Substance use”, and “Traumatic Stress” (44%), followed by “Access to quality health care and other services” and “Geographic distance and other barriers to services” (37%), and finally “Diet/Nutrition/Exercise” and “Education” (15%) (Fig. 4). Respondents were asked to select three programs that are, or could, improve the health of their community the most. The response options included categories of evidence-based programs identified in the scoping review as well as an “Other” category in which participants could enter their recommendations. The most frequently selected options included “Community partnerships providing substance use treatment, including opioid use disorder medications and residential care” (15%), “providing mental health treatment using video visits” (13%), “Community partnerships providing health services for children and families” (13%), and “Informing people about how to prevent and manage diabetes” (12%). Other participant-provided responses included six entries related to improving medical services by increasing interdisciplinary and specialty care for low-income patients, women, and seniors, and one for promoting pharmacy acceptance of Medication Assisted Treatment for Opioid Use Disorder.

### Phase 3: Regional Health Improvement Plan

Regional stakeholders reviewed the results of the community context assessment and provided revisions to the draft document in a collaborative strategic planning process. These stakeholders recommended three domains be prioritized for immediate intervention, and five strategic programs were selected as the most aligned with community-identified priorities, feasible in the local rural context, and supported by the public health literature.

The *2023–2024 Blueprint for Health Improvement and Health-Enabled Prosperity*^21^ recommended the following programs for priority implementation:

- Increasing access to local health care by:
- Establishing telehealth access points at, and transit support to, rural clinics, libraries, community centers, senior centers, pharmacies, and others.
- Increasing rural school-based health services in-person and via telehealth for students and staff.
- Reduce substance use, trauma, and poverty by:
- Providing integrated mental and behavioral health treatment, including trauma-informed therapy and medication management, both in-person in an outpatient capacity and via telehealth.
- Coordinating peer recovery specialist support with a trauma informed lens to help those in active addiction and long-term recovery achieve personal goals, both in-person and via telehealth.
- Promote healthy diet/nutrition and exercise through:
- Nutrition management and physical activity education and support, including outpatient diabetes management, both in-person in an outpatient capacity and via telehealth.

The SWVHA reviewed and approved the programmatic domains and strategies in June 2024. The SWVHA initiated a process by which the recommendations described in the Blueprint could be implemented. This process invited submissions from regional stakeholders in response to a formal RFP in which proposals were required to specify the priority program domains to be addressed. Prospective grantees were instructed to recommend both process and outcome criteria by which their proposed projects would be evaluated. The SWVHA evaluated all proposals received based on alignment with strategic priorities, organizational capacity, sustainability, and elected to sponsor seven projects related to one or more of the three domains. These projects will be implemented and evaluated over the next 12 months.

## DISCUSSION

This iterative, mixed-method CHNA informed the identification and prioritization of regional health issues and supported the development of a strategic planning framework aimed at improving long-term rural health outcomes in VA’s three westernmost health districts. Integrating a literature-based scoping review to inform the community survey design is a relatively novel approach that strengthened the alignment between community perspectives and evidence-based strategies.

While many findings aligned with known regional disparities, such as high rates of poverty, chronic illness, and substance use, there were also noteworthy nuances. The strong resistance among residents to the term “determinants,” for example, reflects a powerful sense of agency and identity that can influence program messaging and community buy-in. Similarly, the intensity with which rurality was framed as both a geographic and cultural barrier to care provides insights for future service design beyond transportation logistics alone.

This CHNA also confirmed prior research on the importance of addressing interconnected root causes. Community members repeatedly emphasized the linkages between many of the root causes of regional health disparities, such as poverty, trauma, and substance use. These findings may help explain why population health programs addressing just one of these root causes in isolation may fail to achieve a lasting health impact.

Several limitations should be recognized. First, the survey relied on a self-selected, non-random sample, which may over-represent more engaged or health-literate residents, particularly health professionals. Second, although efforts were made to include a broad cross-section of counties, some geographic and demographic groups may remain underrepresented. Third, the timeline for data collection and strategy development was relatively compressed, potentially limiting the depth of community engagement.

Considering these limitations, the process proved highly effective in producing a community-validated, place-based, and evidence-informed strategic framework. The integration of public input and scientific evidence supported stakeholder trust and accelerated regional adoption efforts. Future iterations may consider extending the outreach window, incorporating more structured consensus-building methods, or linking community feedback to longitudinal health outcomes.

## IMPLICATIONS

The latest *Blueprint* presented community health priorities and recommended a multi-sectoral set of programs with established protocols and metrics for implementation. These recommendations were adopted by the SWVHA, which has allocated resources to support projects implementing these priority strategies. This iterative effort was crucial in the development and evaluation of integrated and effective population health interventions in the region. Future regional community health assessments will evaluate progress on these projects and recalibrate regional efforts in response to evolving local needs and priorities. In this way, the regional *Blueprint* becomes a living document, building a stronger and healthier future for the residents of Appalachian Virginia.

The *2023–2024 Blueprint* translated resident-identified priorities into actionable recommendations that align with evidence informed practices and supported by recognized evaluation metrics. The SWVHA’s adoption of these recommendations and allocation of implementation funding to seven local projects demonstrates a strong regional commitment to health improvement.

The approach described here serves as a model for other regions seeking to blend grassroots engagement with evidence-based planning and implementation. As rural communities across the U.S. face exacerbating health and infrastructure challenges, this model offers a replicable pathway prioritizing fostered regional resiliency, community ownership and advocacy, and measurable evaluative impact. Future CHNAs will be essential in assessing project outcomes, refining aligned regional goals, and the iteration of community priorities in response to changing needs and health care landscapes.

**SUMMARY BOX**

**What is already known about this topic?**

Residents of Virginia’s (VA’s) Appalachian Region experience higher rates of preventable health outcomes compared to state and national averages. There is a paucity of information on the root causes of these health disparities, or how to effectively address them.

**What is added by this report?**

This iterative, mixed-method community health needs assessment informed the identification and prioritization of regional health issues and supported the development of a strategic planning framework aimed at improving long-term rural health outcomes in VA’s three westernmost health districts. As many root causes of regional health disparities are interconnected, so too were our efforts to address them.

**What are the implications for future research?**

This mixed method design supported the community-based development of population health interventions in the region. Future regional community health assessments will evaluate progress on these projects and recalibrate regional efforts in response to evolving local needs and priorities.
